# Supplementary material for: Relaxation dynamics of glasses along a wide stability and temperature range
Source: Sci Rep. 2016 Oct 21;6:35607. doi: 10.1038/srep35607 (PMC5073287; doi:10.1038/srep35607)
Supplement: Supplementary Information [file srep35607-s1.pdf]

**Supplementary Information.**

**Relaxation dynamics of glasses along a wide stability and temperature range**

C. Rodríguez-Tinoco, J. Ràfols-Ribé, M. González-Silveira, J. Rodríguez-Viejo

Physics Department, Universitat Autònoma de Barcelona, 08193 Bellaterra, Spain.

## Derivation of relaxation and transformation times.

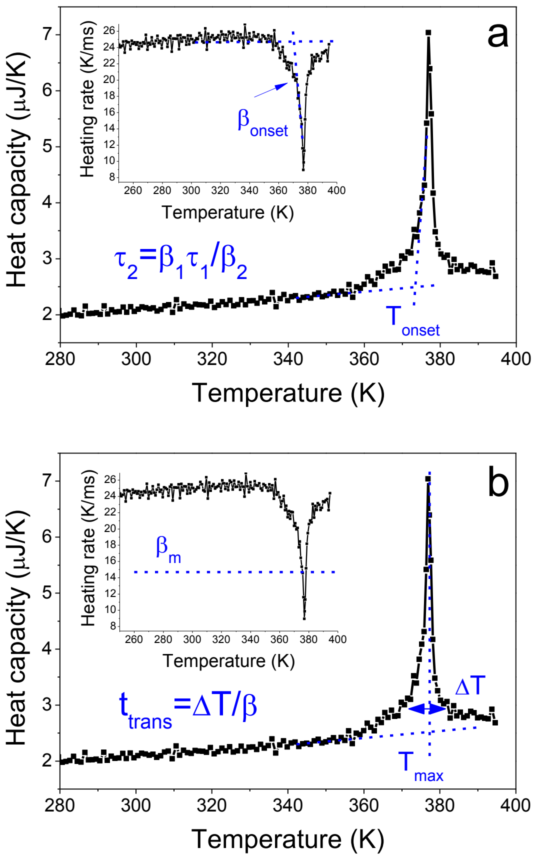


**Supplementary Figure 1. Schema of the calculation of relaxation and transformation times from heat capacity data.** a) in the first approach, we use the expression $\boldsymbol{\tau_1\beta_1 = \tau_2\beta_2}$ to obtain the value of glass relaxation time from the heating rate of the experiment, assigning this value to the onset temperature. b) in the second approach, we calculate the transformation time from the width of the transformation peak and the midpoint value of the heating rate, assigning it to the temperature at the maximum of the transformation peak.

## Comparison between procedures to determine relaxation and transformation times

In the supplementary Fig. 2 we show the comparison among different methods to infer the value of relaxation time of a glass with a particular stability and heated at a given rate. In the Supplementary Fig. 2a, we compare the transformation time calculated, on one hand, using the expression referred in the main text, $t_{trans}(T_{max}) = \Delta T/\beta$, and, on the other, the transformation time directly measured from an isotherm measure at the same temperature performed in a conventional DSC. The transformation time is considered to be the time elapsed from the beginning of the isotherm process to the moment at which the power output of the DSC is constant. The transformation time obtained from the two methods, 182 and 150 seconds respectively, are fairly comparable.

On the other hand, we can derive the relaxation time of a glass at the onset of the transformation measured at a given rate from the well-known expression $\tau_1\beta_1 = \tau_2\beta_2$, taking as reference values $\tau_1 = 100\ s$ and $\beta_1 = 0.167\ K/s$. In particular, for an ultrastable glass measured at $\beta_2 = 0.033\ K/s$, $\tau_2 = 506\ s$. We can compare this value to the one obtained by performing an isothermal measurement at the same temperature ($T_{on} = 332\ K$), 550 s, in fair agreement with the previous result (Supplementary Fig. 2b).

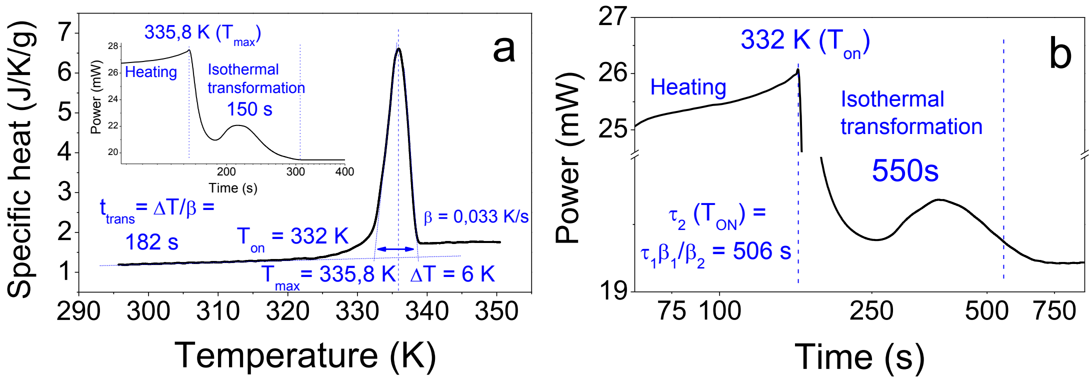


**Supplementary Figure 2. Comparison between procedures to determine relaxation times**. a) DSC scan of an IMC glass deposited at $T_{dep}$ = 266 K, heated at 0.033 K/s. From the width of the peak and the heating rate, the transformation time is inferred as indicated and assigned to the temperature where the maximum of the peak appears. In the inset, a DSC isotherm on an equivalent sample performed at the temperature of the maximum of the peak is shown. From that measurement, we find the transformation time of the sample at that temperature. From the two measurements, we find that both methodologies are approximately equivalent. b) a DSC isotherm on an equivalent sample performed at the onset temperature of the transformation is shown. The transformation time is fairly equivalent to the relaxation time inferred using the expression $\boldsymbol{\tau_1 \beta_1 = \tau_2 \beta_2}$, as explained in the text.

In Supplementary Fig. 3 we plot the relaxation time (a) and transformation time (b) calculated for glasses with different stability and measured at different heating rates, together with the structural relaxation time published for IMC supercooled liquid. The fitting of these data using equation (2) yields similar curves, as seen in Supplementary table 1. Also, the limiting fictive temperature values obtained by fitting the experimental points shown in Supplementary Fig. 3 a and b, and also obtained from the combined data shown in Fig. 1a, yields similar results, as seen in Supplementary Table 2.

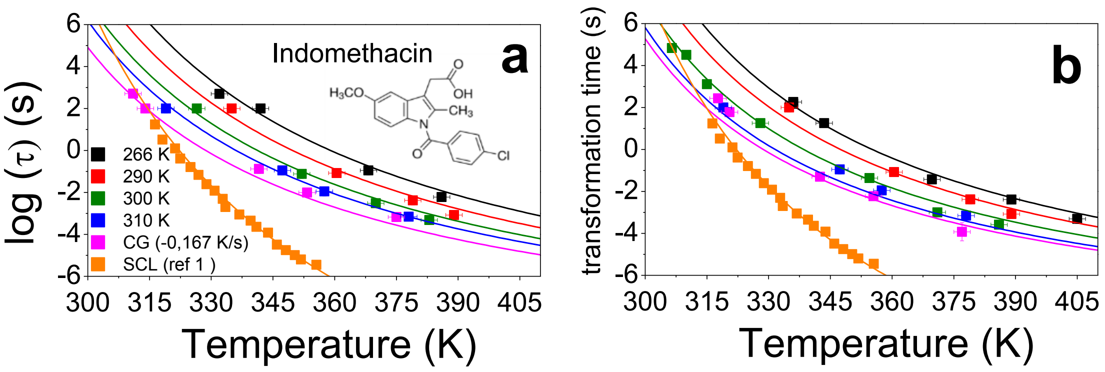


**Supplementary Figure 3. Comparison between the adjustment of relaxation and transformation times with equation (2).** Relaxation (a) and transformation (b) times of IMC glasses with different stability measured at different heating rates and structural relaxation time of supercooled IMC liquid from ref[1]. The fits correspond to equation (2). The fit parameters are shown in supplementary table 1 and 2.

From these observations, we can infer that: i) the relaxation time of glasses, calculated using the expression, $\tau_1\beta_1 = \tau_2\beta_2$, and that of the supercooled liquid can be described using the same empirical relationship, and ii) the similitude between the fitting parameters obtained using the expression above for the relaxation times and those determined from the transformation times, is indicative of the similarity between the two concepts, at least in the experimental conditions under which our experiments were performed. For all this, we consider both measurements as representative of the same magnitude, the relaxation time of the glass.

The agreement between the nominal limiting fictive temperature of the measured glasses and $T_f'$ obtained by fitting the experimental data using equation (5) can also be seen from the data shown in Supplementary Table 2.

**Supplementary Table 1**. Fitting values using equation (2) of the main text. τ refers to relaxation times calculated from $\tau_1\beta_1 = \tau_2\beta_2$ assuming $\tau_1$ = 100 s for $\beta_1$ = 0.167 K/s, $t_{trans}$ refers to transformation times. τ + $t_{trans}$ refers to the fitting using all data. The later are used in the main text.

| Indomethacin | | $T_0$ (K) | A ($K^{-1}$) | B | $\tau_{g0}$ (s) | $\tau_0$ (s) | D |
|---|---|---|---|---|---|---|---|
| Fitted data | τ | 228.59 | -0.107 | 45.62 | 1.91e-12 | 5.75e-23 | 21.16 |
| | $t_{trans}$ | 229.86 | -0.105 | 44.8 | 2.57e-12 | 1.07e-22 | 20.66 |
| | τ + $t_{trans}$ | 230.54 | -0.106 | 44.93 | 2.69e-12 | 8.9e-23 | 20.55 |

**Supplementary Table 2.** Values of limiting fictive temperature for IMC glasses resulting from the various fittings using equation (2) and equation (5) compared to nominal values, $T_{f,\ nominal}$, obtained by integration of the heat capacity curves.

| Indomethacin | | | | | | |
|---|---|---|---|---|---|---|
| $T_{dep}$ (K) | | | 266 | 290 | 300 | 310 |
| $T_f'$ fit (K) (using eq. (2)) | Fitted data | τ | 284.5±1.5 | 293.3±1.2 | 303.7±1 | 309.3±1.2 |
| | | $t_{trans}$ | 284.9±1.4 | 293.6±1.5 | 303.9±1 | 309.4±1.1 |
| | | τ + $t_{trans}$ | 285.2±1.4 | 293.8±1.4 | 304±1 | 309.6±1.1 |
| $T'_{f,\ nominal}$ (K) | | | 279±2.5 | 289±2.5 | 301±2.5 | 311.5±2.5 |
| **Toluene** | | | | | | |
| $T_{dep}$ (K) | | | 111 | 113 | 116 | |
| $T_f'$ fit (K) | | τ + $t_{trans}$ | 107.2±1.3 | 111.4±1.1 | 118.3±0.8 | |
| $T'_{f,\ nominal}$ (K) | | | 111±2.5 | 113±2.5 | 116±2.5 | |

## Representation of relaxation data as a function of the inverse of temperature

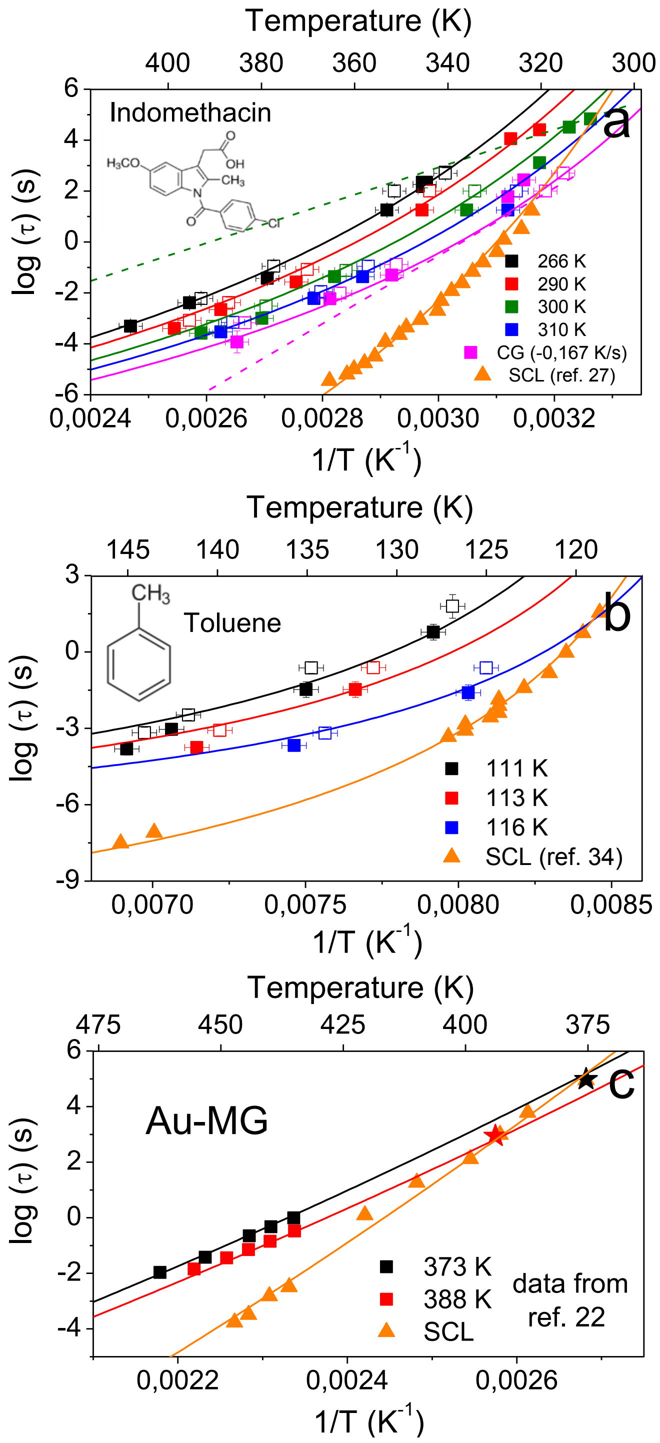


**Supplementary Figure 4**. Representation of the relaxation data shown in Figure 1 as a function of 1/T, to better visualize the non-Arrhenius dependence of the glass relaxation time in a sufficiently extended temperature range. References from the main text.

## Derivation of D and $\tau_0$ from equation (2)

In supercooled liquid, $T_f = T$ at all the temperatures. In this case, equation (1) and (2) should coincide. Therefore,

$$\tau_{g0} e^{\frac{\xi(T) T_0}{(T - T_0)}} = \tau_0 e^{\frac{D T_0}{(T - T_0)}}$$

Taking natural logarithms and isolating, $\xi(T)$, we obtain that,

$$\xi(T) = -\frac{1}{T_0} \ln\left(\frac{\tau_{0g}}{\tau_0}\right) T + \left(D + \ln\left(\frac{\tau_{0g}}{\tau_0}\right)\right)$$

Assuming the expression, $\xi = AT_f + B$, we see that,

$$A = -\frac{1}{T_0} \ln\left(\frac{\tau_{0g}}{\tau_0}\right)$$

$$B = \left(D + \ln\left(\frac{\tau_{0g}}{\tau_0}\right)\right)$$

From where we obtain the equations (3) and (4) for D and $\tau_0$.

## Calculation of density as a function of stability and temperature

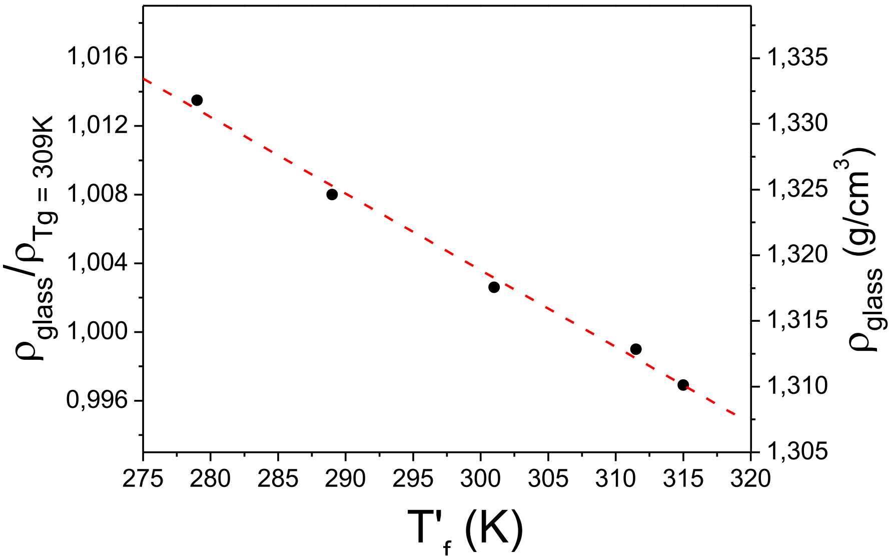


**Supplementary Figure 5. Density of IMC glasses with different stability at 293 K extracted from ref**[2]. In the left axis, data is represented with respect to the density of a glass cooled at 1 K/min ($\mathbf{T_f'}$ = 309 K), as shown in the reference. Right axis has been stablished after the consideration that, for $\mathbf{T_f'}$ = 315 K (conventional glass), the density is 1.31 g/cm$^3$. Transformation from $T_{dep}$ data to $\mathbf{T_f'}$ has been performed according to the relationship between the two quantities, as shown in ref[3].

**Supplementary table 3.** Values of density and thermal expansion coefficients used for each glass and for the supercooled liquid in equation (5) to construct the Supplementary Fig. 4.

| $\mathbf{T'_{f,nominal}(K)}$ | $\boldsymbol{\rho_0\left(\frac{g}{cm^3}\right)}$ **at 293 K** | $\boldsymbol{\alpha_T(K^{-1})}$ **x** $\mathbf{10^{-4}}$ |
|---|---|---|
| **279** | 1.332 | 1.39 |
| **289** | 1.325 | 1.374 |
| **301** | 1.318 | 1.354 |
| **311.5** | 1.313 | 1.33 |
| **315 (SC -10 K/min)** | 1.31 | 1.32 |
| **SCL** | 1.307 (at 315 K) | 5.69 |

**Derivation of scaling parameter from relaxation time data of supercooled IMC liquid**

In Supplementary Figure 6a, we plot the relaxation time of supercooled indomethacin liquid as a function of temperature and at different isobars, measured by Wojnarowska et al.[1]. In order to obtain the scaling parameter, we fit this experimental data using the equation 5 from the main text. The specific volume as a function of temperature at different isobars is extrapolated from the PVT data reported by Adrjanowicz et al.[4].

In Supplementary figure 6b we plot the $\log T_g$ vs $\log v_g$, where $T_g$ and $v_g$ refer to the temperature and specific volume of the system at the transition from liquid to glass, obtained from the reported PVT data[4]. From the slope of this curve, the scaling parameter can also be found, according to[5]

$$\log T_g = A - \gamma \log v_g$$

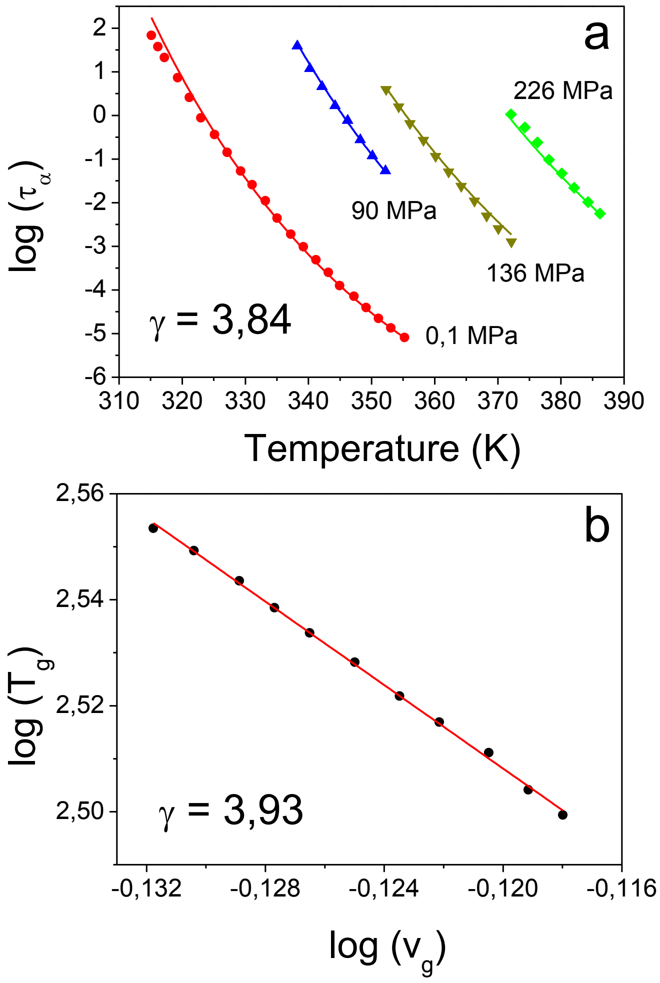


**Supplementary figure 6.** a) Relaxation time of supercooled IMC at different isobars. The curves are fitted using equation 5 from the main text, yielding a scaling parameter of 3.84. b) $\mathbf{logT_g}$ vs $\mathbf{logv_g}$, where $\mathbf{T_g}$ and $\mathbf{v_g}$ refer to the temperature and specific volume of the system at the transition from liquid to glass. All experimental data have been extracted from reported results[1,4].

<u>**Alternative calculation of scaling parameter from glass relaxation time data**</u>

From the relaxation data plotted in Figure 2b in the main text, we obtain $T_g$ as the temperature at which the relaxation time of the glass equals 100 s. At this temperature, the specific volume of the glass is $v_g$. From the slope of the representation shown in Supplementary figure 7, we can calculate the scaling factor, according to

$$\log T_g = A - \gamma \log v_g$$

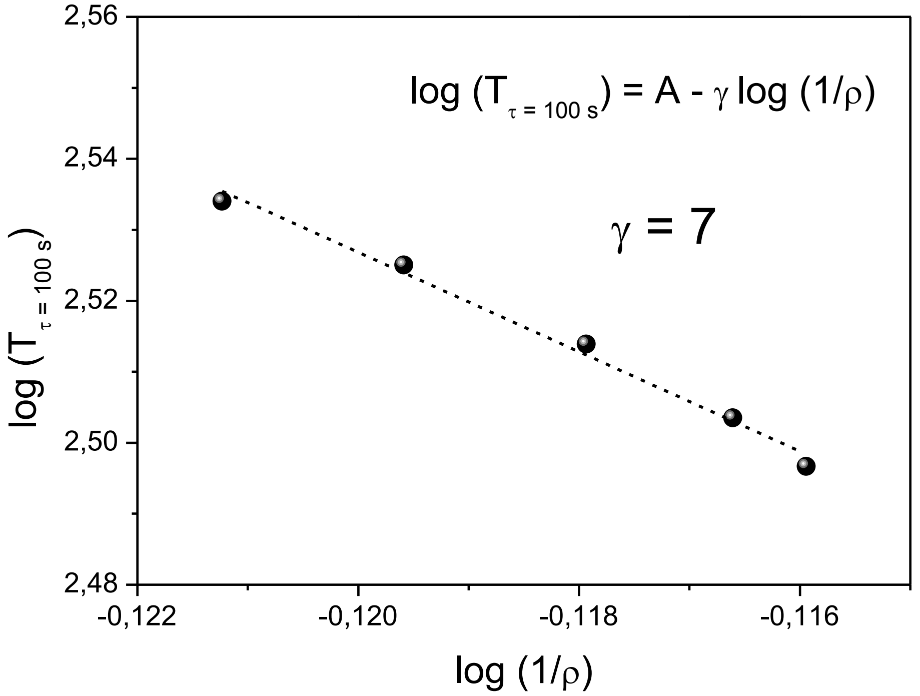


**Supplementary figure 7**. **$\log T_g$** vs **$\log v_g$**, where **$T_g$** and **$v_g$** refer to the temperature and specific volume of the system when the relaxation time equals 100 s. The slope of the fitted curve corresponds to the scaling parameter.

We note the similitude between the scaling parameter found from the fitting of the relaxation time using equation 5 in the main text ($\gamma_{glass} = 6.53$) and using the approach in Supplementary figure 7 ($\gamma_{glass} = 7$).

## SUPPLEMENTARY REFERENCES


1. Wojnarowska, Z. *et al.* Broadband dielectric relaxation study at ambient and elevated pressure of molecular dynamics of pharmaceutical: indomethacin. *J. Phys. Chem. B* **113,** 12536–45 (2009).

2. Dalal, S. S., Fakhraai, Z. & Ediger, M. D. High-throughput ellipsometric characterization of vapor-deposited indomethacin glasses. *J. Phys. Chem. B* **117,** 15415–25 (2013).

3. Rodriguez-Tinoco, C., Gonzalez-Silveira, M., Rafols-Ribe, J., Lopeandia, A. F. & Rodriguez-Viejo, J. Transformation kinetics of vapor-deposited thin film organic glasses: the role of stability and molecular packing anisotropy. *Phys. Chem. Chem. Phys.* **17,** 31195–31201 (2015).

4. Adrjanowicz, K., Grzybowski, A., Grzybowska, K., Pionteck, J. & Paluch, M. Toward Better Understanding Crystallization of Supercooled Liquids under Compression: Isochronal Crystallization Kinetics Approach. *Cryst. Growth Des.* **13,** 4648–4654 (2013).

5. Roland, C. M., Hensel-Bielowka, S., Paluch, M. & Casalini, R. Supercooled dynamics of glass-forming liquids and polymers under hydrostatic pressure. *Reports Prog. Phys.* **68,** 1405–1478 (2005).
